# Impact of Unmet Social Needs on Access to Breast Cancer Screening and Treatment: An Analysis of Barriers Faced by Patients in a Breast Cancer Navigation Program

**DOI:** 10.1245/s10434-025-17608-y

**Published:** 2025-07-02

**Authors:** Grace Keegan, Joseph Ravenell, Angelena Crown, Charles DiMaggio, Kathie-Ann Joseph

**Affiliations:** 1 Department of Medicine, NYU Grossman School of Medicine, New York, NY USA; 2 True Family Women’s Cancer Center, Swedish Cancer Institute, Seattle, WA USA; 3 Department of Surgery, NYU Grossman School of Medicine, New York, NY USA; 4 Departments of Surgery and Population Health, NYU Grossman School of Medicine, New York, NY USA; 5 Surgery, Weill Cornell Medicine, New York, NY USA

**Keywords:** Health disparities, Screening barriers, Unmet social needs, Breast cancer disparities, Patient navigation, Cancer screening, Cancer navigation

## Abstract

**Background:**

Unmet structural and social needs create barriers to breast cancer screening and treatment. The impact of the intersection of these barriers on screening participation and timeliness of breast cancer care remains poorly understood.

**Methods:**

People identifying as women participating in a breast cancer navigation program for screening or treatment were included. Patient navigators administered survey questions that addressed potential barriers to care access using the Health Leads Screening Toolkit. Odds ratios were calculated for unadjusted bivariate associations, and Cox proportional hazards were used to examine the relationship between barriers and time to treatment.

**Results:**

A total of 2804 women (mean age, 53 years) enrolled in navigation for screening or cancer treatment participated in the survey about barriers to care. Of those, 435 (16%) reported unstable housing, 610 (23%) reported poor health literacy, and 164 (6%) reported feeling depressed. Limited transportation was significantly associated with unstable housing (odds ratio [OR] = 26.5, 95% confidence interval [CI] 19.9–35.4, *p* < 0.00001), poor health literacy (OR = 11.5, 95% CI 9.3–14.2, *p* < 0.0001), and depression (OR = 2.9, 95% CI 2.1–4.0, *p* < 0.00001). Individual barriers were not associated with a longer time to treatment, but an increasing number of barriers was associated with a longer time to treatment (Coef = 0.9, *p* < 0.05).

**Conclusions:**

Compounding structural and social barriers limit participation in breast cancer screening, and women with increasing unmet social needs face delays in treatment for breast cancer. Navigation programs may help women overcome barriers to care; however, understanding and targeting the intersectionality of unmet needs is essential for targeted interventions through breast cancer care navigation programs to be effective.

Medically underserved populations face unmet social needs that contribute to lower breast cancer screening rates, later-stage cancer presentation, and ultimately greater cancer burden and mortality.^1^ In New York, where 14% of the population lives below the federal poverty line, counties with low socioeconomic status have low screening rates for breast cancer and higher mortality rates.^2–4^ Breast cancer screening rates are also 20% lower among uninsured individuals in New York compared with people with insurance.^5^ Poverty and underinsurance are not the only barriers that contribute to poor health outcomes, as health literacy and mental health are also factors associated with differences in the health of minoritized and underserved populations.^6–8^

Patient outreach and navigation is an effective strategy for reducing barriers to and disparities in proper cancer screening and care for women by increasing patient follow-up and retention in prevention and treatment plans.^9–11^ The integration of telehealth into these programs stimulated by the COVID-19 pandemic has increased the reach of patient navigation, particularly to underserved populations, and provided a solution for reducing time to diagnosis in many hard-to-reach communities.^12^ However, while navigation has proven to be a valuable tool for increasing the uptake of screening and cancer treatment in these populations, women continue to face compounding barriers to breast cancer screening and treatment.^13–16^

Unmet social needs create barriers to screening and preventative care, but the correlations between and additive effect of these barriers has not been previously described.^1,13,17^ The population of medically underserved women approached in the community for participation in our navigation program provides an opportunity for studying the intersectionality of these barriers. We aimed to characterize the interactions between structural and social barriers among women recruited for patient navigation, including patients in the program for screening and patients enrolled for navigation through cancer treatment. We explored the relationship between these barriers and the time to treatment for women with breast cancer.

## Methods

### Participants

Women from medically underserved backgrounds were approached for participation in a breast cancer screening and treatment navigation program through community outreach events and clinics across New York City. Patient navigators are full-time employees hired from the communities from which they recruit patients. They received standardized training on patient education and navigation provided by George Washington University’s online platform. Patients were connected with patient navigators through two pathways: (1) Patient navigators in the community conducted outreach events at community organizations, including churches and mosques, beauty salons, and community health organizations and approached patients eligible for breast cancer screening, and (2) Patients at several community health centers previously diagnosed with cancer were referred to the patient navigation program for one-on-one support through the cancer treatment process.

### Data Collection

Data were collected from March 2021 to October 2023. Patient navigators working with both groups of patients—patients enrolled for screening and patients enrolled for cancer care—administered survey questions that addressed potential barriers to care access. The Health Leads Screening Toolkit was used to develop the survey questions across 14 domains of structural and social needs. Barriers related to transportation, housing, health literacy, and mental health were evaluated. Demographic information was also collected. Of note, race reported in this paper is the primary race reported by the patient, although the survey allowed patients to report multiple races. Time to treatment was calculated as the time between pathological diagnosis and the patient’s first surgery, radiation treatment, or dose of systemic therapy, depending on what the patient received first.

### Barrier Categorization

Barrier data were collected by using responses to yes/no questions except the question about health literacy. The barrier labeled “transportation” was collected from the survey question, “In the last 12 months, have you ever had to go without health care because you didn’t have a way to get there?” The barrier labeled “housing” was collected from the survey question, “Are you worried that in the next 12 months you may not have stable housing?” The barrier labeled “health literacy” was collected from the survey question, “How often do you have a problem understanding what is told to you about your medical condition?” (For this question, responses were Always, Often, Sometimes, Occasionally, and Never). The barrier labeled “depression” was collected from the question, “Over the last two weeks, have you been bothered by feeling down, depressed, or hopeless?” Other barriers included in the descriptive analysis were collected as follows: “affording medications” from, “In the last 12 months did you skip medications to save money?”; “disability” from, “Do you have a disability that prevents you from accepting any kind of work during the next six months?”; “childcare” from, “Do problems getting childcare make it difficult for you to work or study”; and “immigration” from, “In the past 12 months, have you needed to see a doctor but did not because of your immigration status?” Of note, all barriers questions were asked at the first point of contact with the patient, so do not reflect barriers resulting from receiving care.

### Data Analysis

The responses were aggregated and analyzed. Descriptive analyses were conducting in the R statistical software program and consisted of frequencies, proportions, standard deviations and associated 95% confidence intervals.^18^ Comparisons of continuous variables were based on *t*-tests, categorical variables were compared by using chi-square tests as part of the CreateTableOne function of the R.^19^ Unadjusted odds ratios for the associations were calculated by using the epitab function of the R epitools package.^20^ Odds ratios were calculated for the most commonly reported barrier (transportation) versus the next five most commonly reported barriers among the study population (housing, health literacy, depression, childcare, and affording medications). The barriers faced by screening patients were compared with cancer patients. The most common barriers overall (transportation, housing, health literacy), and the most common barriers for cancer patients (depression and disability) were individually evaluated against time to treatment using Cox proportional hazards. The total number of barriers was also evaluated against time to treatment. Hazard ratios (HR) are reported for differences in time to treatment with nonoverlapping 95% confidence intervals. For the time to treatment analysis, health literacy barriers were dichotomized to Yes (Responses = Always, Often) and No (Responses = Sometimes, Occasionally, Never).

## Results

A total of 2,804 women participated in the survey about barriers to accessing preventative screening for breast cancer. The demographics of respondents are displayed in Table 1. The mean age of respondents was 52.7 years. The most frequent self-reported races were Black (*n* = 941, 33.6%), Hispanic/Latinx (*n* = 554, 19.8%), and White (*n* = 383, 13.7%); the self-reported unemployment rate among the study population was 15.8% (*n* = 419).

**Table 1:** Demographics and barriers reported by all patients in navigation

	No. patients (%)
Demographic	
Total patients	2804
Race	
Black or African American	941 (33.6)
Hispanic or Latino or Spanish	554 (19.8)
White	383 (13.7)
Asian	78 (2.8)
American Indian or Native American	41 (1.5)
Pacific Islander or Native Hawaiian	16 (0.6)
Unknown	661 (23.6)
Prefer Not to Answer	130 (4.6)
Unemployed	419 (15.8)
Age (mean (SD))	52.7 (11.7)
Barriers	
Transportation	762 (28.8)
Housing	435 (16.4)
Health literacy	
Always	325 (12.3)
Often	285 (10.8)
Sometimes	285 (10.8)
Occasionally	371 (14.1)
Never	1373 (52.0)
Depression	164 (6.2)
Affording medications	144 (5.4)
Disability	109 (4.2)
Childcare	264 (10)
Immigration	124 (4.7)

Transportation was the most commonly reported barrier among the patient population surveyed; 28.8% (*n* = 762) of all patients reported having difficulty seeking medical care due to transportation challenges. Unmet housing needs were reported by 16.4% (*n* = 435) of all patients in the program. Similarly, 12.3% (*n* = 325) of women in the study reported always having difficulty understanding medical information, whereas just 52% (*n* = 1373) reported never facing health literacy challenges. Additionally, 6.2% (*n* = 164) of patients reported feeling depressed, 5.4% (*n* = 144) of patients indicated they skipped medications because they had difficulty affording them, 4.2% (*n* = 109) reported a disability, 10% (*n* = 264) reported having childcare concerns that prevented them from completing daily activities, and 4.7% (*n* = 124) indicated that immigration concerns prevented them from seeking medical care. Barriers reported by all patients in the combined navigation programs are reported in Table 1.

The correlations between patients’ responses to facing transportation barriers and facing barriers related to unstable housing, low health literacy, depression, difficulty affording medications, having a disability, childcare challenges, and immigration concerns are listed in Table 2. Race was significantly correlated with responding “yes” to facing transportation barriers (*p* < 0.001), with women identifying as Black having the highest rate of reporting “yes” versus “no” to transportation challenges preventing them from getting medical care (38.3% vs. 32%). There was a strong association between having unstable housing and facing transportation barriers that limit access to healthcare (odds ratio [OR] = 26.5; 95% confidence interval [CI] 19.9–35.4, *p* < 0.00001). Patients reporting symptoms of depression were three times more likely to have gone without medical care owing to difficulty traveling to medical care (OR = 2.9; 95% CI 2.1–4, *p* < 0.00001). There was also a strong association between having difficulty understanding medical information and having difficulty traveling for medical care (OR = 11.5; 95% CI 9.3–14.2, *p* < 0.0001). The odds of having difficulty affording medications among those who had transportation barriers was 11.5 (95% CI 7.5–17.5, *p* < 0.0001), and women with transportation needs were 2.3 times more likely to have childcare needs impacting their ability to work or study (95% CI 1.8–3.9, *p* < 0.0001). Having a disability (*p* < 0.001) and facing concerns with childcare (*p* < 0.001) were both significantly correlated with responding “yes” to having transportation challenges.

**Table 2:** Correlations between transportation barriers and other unmet social needs

	Patient response to transportation barriers		*p*	Odds ratio
	Yes	No		
	Number (%)	Number (%)		
Demographics				
Race				
Black or African American	292 (38.3)	601 (32)	<0.001	
Hispanic or Latino or Spanish	369 (19.6)	157 (20.6)		
White	108 (14.2)	222 (11.8)		
Asian	11 (1.4)	60 (3.2)		
American Indian or Native American	24 (3.1)	14 (0.7)		
Pacific Islander or Native Hawaiian	9 (1.2)	7 (0.4)		
Unknown	66 (8.7)	578 (30.7)		
Prefer not to say	95 (12.5)	30 (1.6)		
Age (mean (SD))	53.1 (11.9)	52 (11.3)	<0.05	
Barriers				
Housing	365 (48)	63 (3.4)	<0.001	19.9
Health literacy				
Always	220 (28.9)	104 (5.6)	<0.001	11.5
Often	204 (26.8)	79 (4.3)		
Sometimes	160 (21.1)	124 (6.7)		
Occasionally	155 (20.4)	214 (11.6)		
Never	21 (2.8)	1331 (71.9)		
Depression	85 (11.3)	77 (4.2)	<0.001	2.9
Affording medications	113 (14.9)	28 (1.5)	<0.001	11.5
Childcare	123 (16.2)	141 (7.6)	<0.001	2.3
Disability	52 (7.1)	56 (3.1)	<0.001	
Immigration status	88 (11.7)	35 (1.9)	<0.001	

There were significant differences in the barriers faced by patients being navigated for cancer screening versus patients with an already diagnosed cancer referred to the navigation program for treatment, as shown in Table 3. Patients undergoing screening were, on average, younger than cancer patients (mean age 54.7 vs. 57.4, *p* < 0.001). Patients enrolled for screening were more likely to face transportation barriers (26.2% vs. 7.4%, *p* < 0.001), housing instability (14.6% vs. 8.3%, *p* < 0.001), poor health literacy (7.8% of screening patients reported “always” having health literacy challenges vs. 1% of the cancer group, *p* < 0.001), and difficulty affording medications (5% vs. 1.5%, *p* < 0.05) compared with patients enrolled in the navigation program undergoing cancer treatment. Patients enrolled for cancer screening were more likely to report depressive symptoms (21.7% vs. 2.9%, *p* < 0.001) and more likely to experience a disability (15% vs. 2.4%, *p* < 0.001).

**Table 3:** Differences in barriers reported by patients navigated for screening versus cancer

	Navigation program		*p*
	Screening	Cancer	
	Number (%)	Number (%)	
Total	1452	213	
Demographics			
Race			
Black or African American	598 (41.2)	55 (25.8)	<0.001
Hispanic or Latino or Spanish	279 (19.2)	29 (13.6)	
White	101 (7.0)	25 (11.7)	
Asian	27 (1.9)	27 (12.7)	
American Indian or Native American	24 (1.7)	2 (0.9)	
Pacific Islander or Native Hawaiian	4 (0.3)	0	
Unknown	380 (26.2)	75 (35.2)	
Prefer Not to Answer	39 (2.7)	0	
Age (mean (SD))	54.7 (9.9)	57.4 (12.9)	<0.001
Barriers			
Transportation	375 (26.2)	15 (7.4)	<0.001
Housing	210 (14.6)	17 (8.3)	<0.05
Health literacy			
Always	111 (7.8)	2 (1)	<0.001
Often	104 (7.3)	6 (3)	
Sometimes	125 (8.7)	16 (8)	
Occasionally	171 (11.9)	58 (29)	
Never	920 (64.3)	118 (59.0)	
Depression	41 (2.9)	43 (21.7)	<0.001
Affording medications	72 (5)	3 (1.5)	<0.05
Disability	33 (2.4)	30 (15)	<0.001
Childcare	66 (4.6)	12 (5.9)	0.53
Immigration	31 (2.2)	5 (2.4)	1.00

Among the 303 patients screened for the cancer care navigation program, information on time to treatment was available for 234 patients (77%). The median time to treatment for all patients was 62 days. Given that transportation, housing, and health literacy were the most commonly reported barriers overall, but depression and disability were more common among cancer patients, we looked at the relationship between these barriers and time to treatment. We found that reporting transportation barriers, housing instability, health literacy, depression, or disability were not significantly associated with time to treatment as individual barriers. However, the total number of barriers was significantly associated with a longer time to treatment with a hazard ratio of 0.9, indicating that each additional barrier was associated with an approximately 10% lower likelihood of expeditious time to treatment compared to a person with no barriers (*p* = 0.02). These findings are summarized in Table 4.

**Table 4:** Relationship between individual and additive barriers and time to treatment

All patients	Median time to treatment (days)		HR	*p*
	63			
Barriers	Yes	No		
Transportation	61 (CI 51,174)	99 (CI 78,127)		
Housing	93 (CI 78,111)	61 (CI 61,234)		
Health literacy	84 (CI 73,106)	82 (CI 56,111)		
Depression	188 (CI 104,234)	76 (CI 63,93)	0.7	0.09
Disability	99 (CI 65, 174)	84 (CI 73,118)		
Number of barriers vs. time to treatment			0.9	0.02*

*HR* hazard ratio

## Discussion

In this study, we found that barriers to preventative breast care and cancer treatment are highly correlated. Specifically, people experiencing transportation challenges are likely to also face housing instability, poor health literacy, depression, difficulty affording medications, disabilities, childcare needs, and immigration status concerns that impede access to health care. Patients receiving screening navigation are more likely to face unmet social needs related to transportation, housing, and medication affordability. In contrast, patients undergoing cancer treatment are more likely to report mental health and disability barriers. While other studies have identified unmet social needs and barriers among patients undergoing navigation for breast cancer treatment, ours is the first to examine the correlations between these barriers, including their compounding effects, and to compare these barriers between screening and cancer patients recruited from the community versus community health centers, respectively.^1,21,22^

### Characterizing the Diverse Needs of Patients Enrolled for Screening and Cancer Treatment

The differences between barriers faced by patients in the navigation program for screening and those in the program for navigating treatment are significant. It is possible that these differences relate to the process by which they are recruited to the navigation program and may indicate even greater barriers than are captured in this study in hard-to-reach, medically underserved communities. Because the patients enrolled for screening are recruited from the community and not from within the medical system, they face greater transportation, housing, health literacy, and medication affordability-related barriers to accessing traditionally delivered medical care. In contrast, the patients enrolled in navigation for cancer treatment are most often referred from community centers and hospitals. Although these patients also report unmet social needs that hinder their access to medical care, the fact that they presented for screening in the past may reveal that these barriers are not as impeding as the needs of patients in the community recruited for screening.

These findings emphasize the importance of reaching patients for cancer screening through navigation programs. Furthermore, through patient navigation recruitment in the community, there may be an opportunity for intervening to provide social services to relieve these barriers to consistent access to care. Our finding that the cancer patients who reported some of the most common barriers overall, transportation, housing instability, health literacy challenges, and depression did not face a longer time to cancer treatment compared to patients who did not report these barriers individually may be an indication of multiple influences on the timeliness of care. At baseline, the cancer patients who have enrolled in navigation may have enough agency to overcome the barriers they report. Navigation may also play a role in helping these patients overcome logistical or educational barriers to eliminate any potential delay in treatment. However, we also found that an increasing number of reported barriers was associated with a longer time to treatment, suggesting the detrimental effect of compounding barriers on access to timely care for breast cancer patients, despite enrollment in navigation.

In contrast, women in the navigation program for cancer treatment are more likely to report depression and a disability that prevents them from working. These findings are consistent with previous studies that demonstrate higher rates of depression and disability among the cancer population.^21,23,24^ This finding suggests the need for supporting all breast cancer patients with mental health services as well as social support for navigating disabilities possibly related to cancer treatment itself.

### Compounding Barriers and Delayed Treatment

The strong correlations between the barriers considered in this study suggest that the same women represent a significant proportion of people reporting these challenges. While women whose social needs are met might overcome these barriers, our system is designed such that women without access to care are inhibited by multifactorial unmet social and structural needs. These findings highlight that intersectional needs contribute to barriers in cancer care, relating specifically to the accessibility of screening. Consequently, the compounding effects of these strongly associated barriers likely contribute to disparities in cancer mortality across populations experiencing these barriers, as patients with less frequent screening often present at later stages of cancer and have worse prognoses.^25^

Interestingly, in a city with extensive public transport systems, our study revealed that transportation barriers remain the most common unmet social need among medically underserved women surveyed in our study. Previous studies have linked transportation challenges with a myriad of poor health indicators, including mammography adherence and stage at cancer presentation.^26–28^ Our study is the first to identify the relationship between transportation challenges and unmet social needs of other realms, including mental health and health literacy, implicating the role that improved transportation access can play in addressing an integral element in the network of challenges faced by these women.

### Towards Intersectional Solutions

Navigation programs such as the one behind this study provide one potential solution to meeting these unmet social needs by intersectional means. This is evident in our finding that cancer patients facing transportation, housing, literacy, and depression barriers do not experience longer time between diagnosis and treatment. However, women facing multiple barriers across the domains examined in this study experience delays in treatment, emphasizing the need for addressing these barriers together instead of in isolation.

The discussion of intersectionality in barriers to cancer care necessitates a discussion of race, as a widespread, systematic social injustice has led to the entanglement of financial, structural, and cognitive barriers with minoritized racial groups. Accordingly, Black women in the United States are more likely to live in poverty, face unstable housing, and have lower health literacy, among a myriad of other unmet social needs.^29,30^ Accordingly, breast cancer mortality to incidence ratios in New York are higher among Black women compared with White women—a pattern also demonstrated in nationwide data.^3,31^ Our study reveals the complex interacting barriers that likely contribute to this trend, where Black women are overrepresented among patients who face transportation barriers (38.3% of those who responded “yes” vs. 33.6% of the study population). This barrier significantly predicts all other psychosocial and socioeconomic barriers discussed in this study. Addressing intersectional barriers to cancer screening and care through breast cancer navigation programs can also be a step toward achieving health equity, especially for minority women.

### Limitations

Our study has limitations. It does not exactly capture the distribution of barriers faced by all women in New York’s neighborhoods, as the survey was only given to women reached by patient navigation programming. Despite extensive community outreach, these programs fall short of providing opportunities for navigation through screening and treatment for all women in need of support. In addition, while all patients come from medically underserved backgrounds, our screening and diagnosed populations come from different referral settings, which may contribute to differences in their barriers as discussed previously. Race data were unknown or not disclosed in over one-quarter of the patients surveyed in the study, making the interpretation of substantial differences in race data across study groups potentially skewed. It is also limited to an urban population, which may limit its external validity, though the most common barriers reported in our study mirror those studied in analogous rural populations.^32^

## Conclusions

Our study reveals that barriers to cancer screening and care comprise intersectional unmet social needs that can make accessing health care insurmountable. Although navigation may contribute to reducing some barriers to care access, women with poor health literacy and compounding barriers face delays in treatment. While patient navigation is one strategy for mitigating the effects of structural barriers to care access for medically underserved populations, comprehensive strategies are needed to address the compounding barriers to care.
